# Dry Feet

**Published:** 1843-06

**Authors:** 


*Dry Feet.*—Capt. John Norton writes thus to the editor of the London Lancet.—After having tried many ways to prevent my boots or shoes from becoming damp by walking the streets in wet weather, I find that a thick varnish, made of shell-lac, dissolved in spirits of wine, and spread, with a brush, on the inside of the shoe, all over the sole, and about half an inch on the upper leather, so as completely to cover the seams, will effectually preserve the feet dry, although the sole of the boot or shoe be less than a quarter of an inch in thickness. This varnish will not soil or adhere to the stocking, even when the feet become heated from much exercise. I have tried this means for some months, and after experiencing the good results, make this communication in the hope that the knowledge of it may prove of general benefit.

